# Outcome of community-initiated treatment of drug-resistant tuberculosis patients in Lagos, Nigeria

**DOI:** 10.1093/trstmh/traa188

**Published:** 2021-01-11

**Authors:** Adebayo M Bakare, Obioha C Udunze, Janet O Bamidele, Amos Omoniyi, Eltayeb Osman, Olusoji J Daniel

**Affiliations:** Damien Foundation Belgium Nigeria project, Lagos State Nigeria; Damien Foundation Belgium Nigeria project, Lagos State Nigeria; Department of Community Medicine and Primary Care, Olabisi Onabanjo University Teaching Hospital Sagamu, Ogun State, Nigeria; World Health Organization, Abuja FCT, Nigeria; Damien Foundation Belgium Nigeria project, Lagos State Nigeria; Department of Community Medicine and Primary Care, Olabisi Onabanjo University Teaching Hospital Sagamu, Ogun State, Nigeria

**Keywords:** adverse drug events, drug-resistant tuberculosis, treatment adherence, treatment outcome

## Abstract

**Background:**

With the improvement in the capacity to diagnose multidrug-resistant/rifampicin-resistant tuberculosis (MDR/RR-TB) patients due to the increased number of GeneXpert machines in Nigeria, the number of patients diagnosed surpassed the bed capacity at MDR-TB treatment centres. Community DR-TB treatment is an important option to improve access to care for MDR/RR-TB patients. However, few studies have determined the outcome of community management of MDR-TB patients, which this study aims to address.

**Methods:**

We conducted a retrospective study of MDR/RR-TB patients initiated on treatment in the community in Lagos, Nigeria, between 1 January 2015 and 31 December 2016. Data were retrieved from DR-TB treatment cards/registers. The treatment outcomes of these patients were assessed at the end of treatment and categorized according to national TB guidelines.

**Results:**

A total of 150 DR-TB patients commenced treatment during the study period. Adherence was 64.7%, with the majority of patients experiencing mild (56.5%) adverse drug events. Treatment was successful in 70% of patients. The only predictor of successful treatment was treatment adherence.

**Conclusions:**

The study shows that community initiation of MDR-TB treatment is feasible and results in a high treatment success rate. Adherence counselling before and during treatment is essential for a favourable treatment outcome.

## Introduction

Multidrug-resistant tuberculosis (MDR-TB) has emerged as a significant public health problem globally and has threatened the progress made in tuberculosis (TB) control over the years. In 2017, the World Health Organization (WHO) estimated that 3.5% of new and 18% of previously treated TB cases have multidrug-resistant or rifampicin-resistant TB (MDR/RR-TB), which translated to an estimated 558 000 new cases and 230 000 deaths. However, only 161 000 new cases were reported globally in 2017 while 131 000 were enrolled in treatment, which is about a quarter of the estimated number of MDR/RR-TB cases.^1^

Nigeria is one of the 30 high MDR-TB burden countries, with an estimated MDR/RR-TB proportion of 4.3% among new cases and 15% among previously treated TB cases in 2018.^2^ A total of 2275 cases were laboratory-confirmed and 1895 (83.3%) patients were enrolled into treatment within the country. With the improvement in the capacity to diagnose rifampicin resistance among TB patients as a result of an increase in the number of GeneXpert machines available in the country, many more MDR/RR-TB patients are being diagnosed. This surpasses the current bed capacity of the MDR-TB wards. Also, some patients refused admission to the treatment centre, thereby making it necessary to initiate treatment in the community.

To address this, the National Tuberculosis and Leprosy Control Programme adopted a community-based treatment approach to the management of MDR/RR-TB to complement the ongoing hospital-based treatment approach.^3^ Community-based treatment for MDR-TB patients has been successfully implemented in Africa and elsewhere. Treatment success in these places ranges from 70 to 83%.^4–7^ Although the pilot community DR-TB treatment programme started in Lagos in 2015, to the best of our knowledge there has been no study evaluating treatment outcomes among this category of MDR/RR-TB patients in Nigeria.

## Materials and methods

Lagos state is located in southwest Nigeria, with a 2016 population projection of about 13 million people according to Nigerian population data from 2006.^8^ Despite being one of the smallest states in the country, it is the commercial nerve centre of Nigeria, with a high influx of people from different parts of the country.

### Study design

A retrospective study design was employed using secondary data of DR-TB patients who were registered in the community-based approach between 1 January 2015 and 31 December 2016 and was followed up for 20 months until completion of treatment. The patients with monoresistance other than rifampicin, pre-extensively drug-resistant TB (pre-XDR-TB) and extensively drug-resistant TB (XDR-TB) patients were excluded from this study. This is because such groups are admitted to hospitals for their treatment and not managed in the community, in line with the National Tuberculosis Control Guidelines. The data for each patient were retrieved from the DR-TB patient treatment card, DR-TB patient treatment progress notes from directly observed treatment short-course (DOTS) providers in the community, the state DR-TB central register, baseline and routine investigation results cards. The data collected included sociodemographic, adverse drug events and human immunodeficiency virus (HIV) status, which was entered into a designated form. Subsequent analysis was done using EPI Info statistical software (Centers for Disease Control, Atlanta, GA, USA). Logistic regression was done to determine the predictors of treatment success and a p-value <0.05 was considered statistically significant.

### Community DR-TB programme Lagos state

Diagnosis of MDR/RR-TB in Lagos state is done using the Xpert MTB/RIF assay. All MDR/RR-TB patients are reported to the local government TB and leprosy supervisor. Sputum samples for culture and drug susceptibility testing (DST) are obtained from each patient. Baseline screening tests such as full blood count, electrolyte and urea, audiometry and liver function tests, are carried out before patients are commenced on second-line TB medications for a minimum of 20 months. The treatment is divided into an intensive phase of 8 months with five drugs (kanamycin, levofloxacin, cycloserine, prothionamide and pyrazinamide) and a continuation phase of 12 months with four drugs (levofloxacin, cycloserine, prothionamide and pyrazinamide).

Under the national programme DR-TB guideline, the oral anti-TB medications are administered daily to patients while the injectables are administered 5 d a week during the intensive phase. The patient is provided with a choice either to travel to the local primary health centre (PHC) close to their residence or the healthcare provider can visit the patient's residence to administer treatment. This allows the healthcare worker to directly observe medications administration all through the duration of treatment. Patients who are very ill and warrant hospital admission are referred to the nearest designated hospital for further management. Patients are reviewed monthly by a council of experts at the state MDR-TB referral centre. During this time the monthly samples for follow-up investigation are collected.

### Treatment outcome of DR-TB patients

The treatment outcome for the cohort of patients registered within the study period was assessed at the end of 20 months of treatment and was assigned to the treatment category according to the National Tuberculosis and Leprosy Control Programme guidelines for the management of drug-resistant TB patients.^9^

- Cure—treatment completed without evidence of failure and three or more consecutive cultures (taken at least 30 d apart after the intensive phase) are negative.
- Completed treatment—treatment completed without evidence of failure but no record that three or more consecutive cultures (taken at least 30 d apart after the intensive phase) were negative.
- Loss to follow up—a patient whose treatment was interrupted for ≥2 consecutive months.
- Treatment failure—treatment terminated or need for permanent regimen change of at least two anti-TB drugs due to lack of conversion by the end of the intensive phase, bacteriological reversion in the continuation phase after conversion to negative or evidence of additional acquired resistance to fluoroquinolones or second-line injectable drugs or due to adverse drug reactions.
- Not evaluated—a patient for whom no treatment outcome is assigned as a result of being transferred out or no record on treatment available to determine the treatment outcome.
- Died—a patient who died for any reason during the course of treatment.

A successful outcome was defined as cure and treatment completed, while unfavourable treatment outcomes were classified as loss to follow-up, failure and died.

Treatment adherence was calculated by dividing the number of days patients used the prescribed medication over the total duration of treatment multiplied by 100%. Good adherence was >95%.

### Adverse events

The Division of AIDS (DAIDS) Table for Grading the Severity of Adult and Paediatric Adverse Events version 2.1 was used to grade the severity of adverse events among patients on treatment. Grade 1 indicates a mild event, grade 2 a moderate event, grade 3 a severe event and grade 4 a potentially life-threatening event.^10^

## Results

A total of 150 DR-TB patients initiated treatment in the community during the study period. Table 1 shows that the majority of the patients were in the age group 25–34 y (36.7%), males (60.7%), had a secondary school education (70.7%) and were single (56%). Table 2 shows that majority had a pretreatment body mass index (BMI) of >18.5 kg/m^2^ (72%). A total of 33 (22%) respondents had ever used alcohol in the past, 8 (5.3%) had ever smoked cigarettes and 14 (9.3%) were HIV positive. The median time to initiate DR-TB treatment after a confirmed diagnosis was 29 d (interquartile range 19 0.0–46.0). Males had a significantly shorter duration before the commencement of treatment compared with females (30.7 ± 21.4 vs 40.6 ± 30.2; p = 0.017).

**Table 1.** Sociodemographic characteristics of the population (N=150)

Variables	Frequency	Percentage
Age (years)		
15–24	35	23.3
25–34	55	36.7
35–44	32	21.4
45–54	20	13.3
>55	8	5.3
Sex		
Male	91	60.7
Female	59	39.3
Educational status		
No formal education	10	6.8
Primary	15	10
Secondary	106	70.7
Post-secondary	19	12.7
Marital status		
Single	84	56
Married	62	41.3
Other	4	2.7
Employment status		
None	46	30.7
Self-employed	89	59.3
Government	15	10.0

**Table 2.** Variables related to behaviour, nutrition, clinical management and HIV status

Characteristics	Frequency	Percentage
BMI (kg/m^2^)		
≤18.5	42	28.0
>18.5	108	72.0
HIV status		
Positive	14	9.3
Negative	136	90.7
Alcohol use		
Yes	33	22.0
No	117	78.0
Ever smoked		
Yes	8	5.3
No	142	94.7
Adverse events (n=115)		
Mild	65	56.5
Moderate	43	37.4
Severe	7	6.1
Drug intake		
Adherent	97	64.7
Non-adherent	53	35.3
Duration (days) between diagnosis and commencement of treatment (n=138)		
≤30	72	52.2
>30	66	47.8

The majority of patients had mild (56.5%) adverse drug events, while only 6.1% experienced severe adverse drug events during treatment. A total of 64.7% of patients were adherent to their medications. Table 3 outlines the various adverse drug events observed among MDR/RR-TB patients during treatment. The most common adverse drug events observed were gastrointestinal symptoms such as nausea, vomiting and loss of appetite. The treatment outcomes of the population are shown in Table 4. A total of 54 (36.0%) completed treatment, 51 (34.0%) were cured, 8 (5.3%) had treatment failure, 19 (12.7%) were lost to follow-up, 14 (9.3%) died and 4 (2.7%) patients were not evaluated. Table 5 showed that adherence to second-line anti-TB medications is the only predictor of successful treatment. Other factors such as sex, age, HIV status, adverse drug events and duration between diagnosis and commencement of treatment were not associated with successful treatment.

**Table 3.** Adverse events observed in MDR/RR-TB patients on treatment

Adverse events	Frequency	Percentage
Gastrointestinal		
Nausea	14	8.3
Vomiting	28	16.7
Loss of appetite	6	3.7
Sensory		
Tinnitus	11	6.7
Loss of hearing	7	4.3
Numbness	7	4.3
Blurred vision	2	1.2
Psychiatric		
Insomnia	15	9.1
Psychosis	1	0.6
Skin		
Itching	8	4.9
Rashes	7	4.3
Musculoskeletal		
Joint pain	31	18.9
Endocrine		
Swollen neck	1	0.6
Systemic		
Dizziness	5	3.0
Fatigue	15	9.1
Respiratory	6	3.7
Chest pain	6	3.7
Total	164	100

**Table 4.** Treatment outcome of MDR/RR-TB patients on second-line medication

Treatment outcome	Frequency	Percentage
Cured	51	34
Treatment completed	54	36
Failure	8	5.3
Died	14	9.3
Lost to follow-up	19	12.7
Not evaluated	4	2.7
Treatment success^a^	105	70
Unsuccessful treatment	45	30

^a^Treatment success is the combination of cure and treatment completed.

**Table 5.** Predictors of successful treatment among MDR-TB patients

Characteristics	Adjusted odds ratio (95% confidence interval)	p-Value
Sex	1.69 (0.23 to 10.50)	0.5
Age	0.99 (0.91 to 1.07)	0.75
BMI (kg/m^2^)	0.96 (0.73 to 1.27)	0.78
HIV	222 (0.01 to 1.00)	0.97
Duration between diagnosis and onset of treatment	0.99 (0.07 to 1.02)	0.99
Drug adherence	79.41 (8.88 to 710.15)	0.0001
Adverse reaction	0.67 (0.23 to 1.96)	0.47
Alcoholism	0.11 (0.01 to 1.44)	0.09
Smoking	15.19 (0.24 to 92.00)	0.19

## Discussion

This study found that 70% of patients initiated on community-based MDR/RR-TB treatment had successful treatment outcomes. This is higher than the 62.9% in a study in India and the 56% reported by the WHO for the overall proportion of MDR/RR-TB patients who were initiated on drugs in 2016.^11^ However, the treatment success was lower than the 77% and 83% reported by other studies in Nigeria.^11,12^ One of the possible reasons for the low treatment success in this study is the high loss to follow-up of 12.7%. The high loss to follow-up may be due to the challenge of tracing patients in the large cosmopolitan area of Lagos state, which has a population of >20 million, compared with the 5% loss to follow-up in Benue state, which has a much smaller population.^13^ The loss to follow-up may result in increased community transmission of MDR-TB. Therefore greater efforts should be made to reduce the number of patients who are lost to follow-up while on treatment.

It should be noted that only 34% of patients had bacteriological evidence of cure. However, this is greater than the 26% reported nationally.^12^ The low cure rate is due to the difficulty in having patients adhere to their monthly outpatient clinic follow-up where sputum samples are collected for TB culture. These operational bottlenecks need to be addressed to improve treatment outcomes and ensure a better quality of life.

More than one-third (35.3%) of patients were not adherent to their medications. Despite the national recommendations on direct observation by healthcare workers throughout the duration of treatment, adherence is still a challenge in a cosmopolitan area like Lagos, where some patients fail to come to the clinic for their drug refill at the stipulated times. The majority of patients who missed their medication were those who travelled to the health facilities to obtain their medication. However, we could not provide conclusive evidence, due to the lack of complete data on this variable for all patients. The national programme may need to explore this further and if found to be the case, the programme may revise the treatment guidelines for healthcare workers to visit the homes of MDR-TB patients for delivery of their medications instead of patients visiting the facility. Suboptimal adherence to MDR-TB treatment causes the development of pre-XDR-TB and XDR-TB, which are expensive, difficult to treat and require the use of more toxic drugs. Medication adherence was the only predictor of a successful treatment outcome (p=0.0001). Therefore adequate counselling before and during treatment is necessary to achieve adherence to medications and subsequently improve favourable treatment outcomes.

The majority of patients in this study had mild gastrointestinal adverse drug events such as nausea, vomiting and fatigue. There was no occurrence of a patient in which the drug was stopped completely as a result of adverse drug reactions. Only 6.1% of patients had severe adverse drug events such as loss of hearing. Similar studies in India and Vietnam reported mild to moderate adverse drug events in 77.3% and 58% of patients, respectively, and severe reactions in 22.7% and 17.73% of patients, respectively.^14,15^ Although adverse drug events were not significantly associated with treatment outcome in this study, some studies have reported better treatment outcomes in patients who experienced adverse drug events.^15,16^ The explanation could be that patients who experienced adverse drug events were likely to be closely monitored by the healthcare providers and this may subsequently result in better adherence to treatment and ultimately a favourable outcome.

The study has some limitations because it utilised secondary data. As a result, the impact of some variables on treatment outcome, such as the proportion of patients who were receiving their drugs at the treatment facility close to their residence and those who were visited by the healthcare workers at home, could not be ascertained. Second, the full results of the first- and second-line DSTs were not available for all patients, and this limited further analysis.

Despite these challenges, this study highlights the feasibility of community-initiated treatment in a large cosmopolitan area like Lagos. With the increasing number of diagnosed MDR-TB patients and fewer facilities available for treatment, it will be beneficial for the national TB programme to strengthen and expand the community-based treatment approach for the management of DR-TB patients.

## Conclusion

The results of this study show that community initiation of MDR-TB treatment is feasible and resulted in a treatment success of 70%, although it was slightly lower than national figures. The majority of the patients experienced mild to moderate adverse drug events and no patient had medications discontinued as a result of severe adverse drug events. Adherence to medications was a predictor of good treatment success. Therefore continued drug adherence counselling before and during treatment is essential for a favourable treatment outcome.

## Data Availability

The data underlying this article were provided by the Lagos state tuberculosis program by permission. Data will be shared upon request to the corresponding author with permission from the Lagos state tuberculosis program.
